# Reproductive Disruption in Wild Longear Sunfish (*Lepomis megalotis*) Exposed to Kraft Mill Effluent

**DOI:** 10.1289/ehp.8130

**Published:** 2005-09-07

**Authors:** Jennifer A. Fentress, Stacy L. Steele, Henry L. Bart, Ann Oliver Cheek

**Affiliations:** 1 Department of Biological Sciences, Southeastern Louisiana University, Hammond, Louisiana, USA; 2 Department of Ecology, Evolution, and Organismal Biology, and; 3 Tulane University Museum of Natural History, Tulane University, New Orleans, Louisiana, USA

**Keywords:** 11-ketotestosterone, endocrine disruption, 17β-estradiol, paper mill effluent, teleost, testosterone, vitellogenin

## Abstract

Worldwide, wild fish living in rivers receiving municipal and industrial discharges may experience endocrine disruption as a result of exposure to anthropogenic pollutants. The purpose of this study was to evaluate the hormonal status of wild fish in a U.S. river receiving unbleached kraft and recycled pulp mill effluent (Pearl River at Bogalusa, LA). We evaluated two alternative hypotheses: the effluent contained constituents that suppressed male and female reproduction, or it contained an androgenic substance that masculinized females. To evaluate the likelihood of fish exposure to effluent, we marked 697 longear sunfish (*Lepomis megalotis*) over a 2-year period; 83% of recaptured fish were found at the site of initial capture, and only one fish migrated from an effluent-receiving site to a reference site. We can reasonably assume that fish captured from an effluent-receiving site are residents, not transitory migrants. To diagnose endocrine disruption, we measured sex steroid hormone [17β-estradiol (E_2_), testosterone (T), and 11-ketotestosterone (11KT)] and vitellogenin (VTG) concentrations in male and female longear sunfish captured at two sites upstream and two sites downstream of the effluent outfall. Kraft pulp mill effluent did not affect male reproductive physiology but did suppress female T and VTG levels when effluent constituted ≥ 1% of river flow. Masculinization was not observed. Longear sunfish in the Pearl River experience moderate reproductive suppression in response to unbleached kraft and recycled pulp mill effluent.

Biologists around the world have accumulated substantial evidence of endocrine disruption in wild fish, particularly in waters receiving sewage treatment plant or kraft mill effluents (KME) (Jobling et al. 1998; McMaster et al. 1996). Wild fish in KME-receiving waters in Canada, Scandinavia, and the United States experience several kinds of reproductive perturbations: reproductive suppression in both sexes (Dube and MacLatchy 2000; Karels et al. 2001; McMaster et al. 1996; Sepulveda et al. 2002), masculinization of females (Bortone and Davis 1994), or male-skewed sex ratios in exposed fry (Larsson and Forlin 2002).

Reproductive suppression includes effects such as delayed sexual maturity, reduced gonad size, suppressed steroid hormone and vitellogenin (VTG) levels, and impaired pituitary hormone release (Karels et al. 2001; McMaster et al. 1996; Sepulveda et al. 2001). In some species, these physiologic effects are accompanied by a limited ability to spawn (lake whitefish, *Coregonus clupeaformis*, and perch, *Perca fluviatilis*) or by reduced fry number and size (largemouth bass, *Micropterus salmoides*), whereas other species are able to spawn normally (longnose sucker, *Catostomus catostomus*; white sucker, *Catostomus commersoni*; and roach, *Rutilus rutilus*) (Karels et al. 2001; Lister and Van Der Kraak 2001; Sepulveda et al. 2003).

Masculinization can include induction of male secondary sex characteristics in females or male-skewed sex ratio in offspring (Howell et al. 1980; Larsson and Forlin 2002). In masculinized mosquitofish (*Gambusia affinis*), females grew a sperm delivery organ, the gonopodium, and displayed malelike courtship behavior toward other females, even though gonadal sex was unchanged and they continued to produce offspring (Howell et al. 1980). A different form of masculinization, male-skewed sex ratio, occurs in the eelpout (*Zoarces viviparus*), a live-bearing marine fish. Females captured in the effluent plume from an elemental chlorine-free bleached pulp mill had broods that were 55–65% male. After a mill shutdown that coincidentally occurred during early eelpout gestation, broods developed with the same 50:50 sex ratio observed at reference sites (Larsson and Forlin 2002).

KME can cause estrogenic effects such as abnormal VTG production in males, but these effects have never been observed in wild populations, only in sexually immature fish exposed under experimental conditions: caged in the effluent-receiving stream for short periods (Mellanen et al. 1999; Soimasuo et al. 1998), exposed in outdoor mesocosms under flow-through conditions (Van Den Heuval and Ellis 2002), or exposed in the laboratory under static renewal conditions (Tremblay and Van Der Kraak 1999).

The purpose of this study was to determine the extent of endocrine disruption in wild fish in a U.S. river receiving unbleached KME. Our objectives were to assess individual site fidelity in order to estimate the likelihood of long-term effluent exposure and to measure sex hormone and VTG levels in males and females upstream and downstream of the mill. We evaluated two alternative hypotheses: the KME contained constituents that suppressed male and female reproduction, or it contained androgenic constituents that masculinized females. We found that the effluent altered female reproductive physiology, suppressing female testosterone (T) and VTG levels.

## Materials and Methods

### Site selection.

The Pearl River originates in northeastern Mississippi and flows into the Gulf of Mexico. Approximately 43% of the river basin is forested land, 10% is marsh and/or swamp, and 27% is agricultural (Mississippi Department of Environmental Quality 2000). Although the basin has little urban or industrial development, several industrial sites are part of the Lower Pearl watershed, including the Bogalusa Mill in Bogalusa, Louisiana [Louisiana Department of Environmental Quality (LDEQ) 2005].

The Bogalusa Mill is an integrated pulp and paper mill that began operation in 1918 and currently manufactures linerboard paper using the unbleached kraft and semichemical processes. The mill pulps 1,814 metric tons/day of softwood and up to 907 metric tons/day of recycled waste paper. Treated process wastewater, sanitary wastewater, and storm water from the processing site are transported via a 3.2-km-long, 1.4-m-diameter pipe to a 92-m-diameter primary clarifier. Clarifier overflow enters a 25.5-ha aerated stabilization basin (ASB), where it is retained for 3.3 days. Water from the ASB enters the Pearl River via a submerged 1.8-m-diameter pipe. The mean daily discharge of effluent during this study (October 2000 through October 2002) was 0.88 ± 0.08 m^3^/sec. We calculated effluent concentration in the river from monthly discharge monitoring reports filed by the Bogalusa Mill with the LDEQ and river flow data from the U.S. Geological Survey (Table 1; LDEQ 2002, USGS 2003). No characterization of resin acids or phytosterols in the effluent is available for this mill.

**Figure 1:**
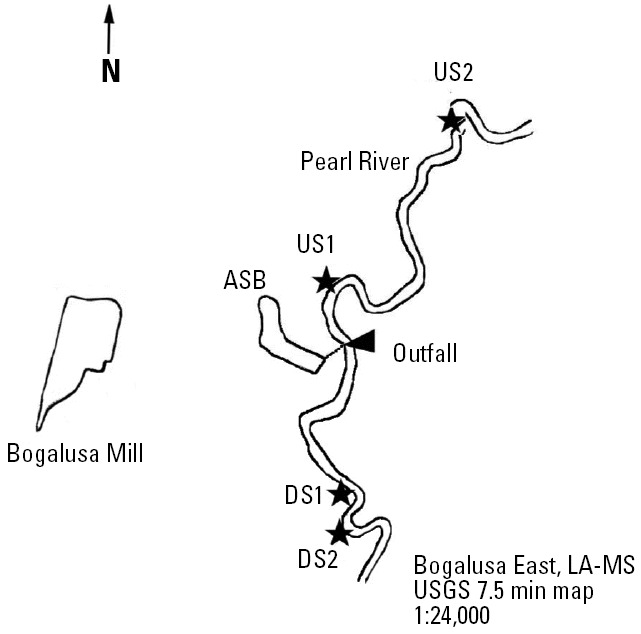
Site locations along the Pearl River near Bogalusa, Louisiana. Outfall, site of wastewater discharge into the Pearl River (river flows south). Map was adapted from the USGS Bogalusa East, LA-MS map, USGS entity ID MPTLA0072PP01, 1997.

We captured and sampled fish at two sites upstream and two sites downstream of the effluent discharge (Figure 1). Sites were chosen based on sunfish (*Lepomis* spp.) abundance. Upstream sites were 2 km (upstream 1, US1) and 5 km (upstream 2, US2) upstream of the discharge site and downstream sites were 1.9 km (downstream 1, DS1) and 2.2 km (downstream 2, DS2) downstream. The downstream sites were close to one another to avoid large differences in effluent dilution. US2 and DS2 (sandbar sites) included shallow sandbars at the mouths of creeks and had slower flow rates relative to the US1 and DS1 sites (streamside sites).

Although we sampled duplicate upstream and downstream sites, these sites are in the same river and must be considered pseudo-replicates. The alternative approach of sampling reference and KME-receiving sites in another river would not provide true replication because the processing, wood furnish, water quality, water flow, and habitat are unique for every mill (McMaster et al. 1996). In our analyses, we compared the US1 and DS1 sites with each other and separately compare the US2 and DS2 sites. Habitat was more similar within each upstream–downstream pair, and US2 and DS2 were sampled less frequently because of the logistical challenges of collecting fish at four sites in a single day.

### Species.

We focused on longear sunfish (*Lepomis megalotis*), the most abundant species of centrarchid in the Pearl River (Gunning and Suttkus 1990). Longear sunfish are fairly sedentary: 80–90% of fish move < 50 m from the site of initial capture (Berra and Gunning 1972; Gunning and Shoop 1963).

Male and female longear sunfish spawn multiple times between April and August, although the exact number of spawns per individual is unknown. As in many sunfish species, male reproductive strategies depend upon body size: large males construct nests in the substrate, court females, and defend eggs. Smaller males are less likely to defend a nest but will streak spawn, darting in to release sperm while a nesting male and female are spawning. Streaking is an opportunistic strategy practiced by all sizes of males, however. Females visit the nests, which may be solitary or grouped in colonies of 2–15 nests, and are more likely to approach and spawn with large males (Dupuis and Keenleyside 1988; Jennings and Phillips 1992).

### Sampling.

All fish used in this study were treated humanely in accordance with a protocol approved by the Southeastern Louisiana University Committee on Use of Humans and Animals in Research. Longear sunfish were captured via electrofishing from October 2000 through October 2002. Fish were sampled twice monthly during the spawning season (within 3 days of the full and new moons) at the US1 and DS1 sites (eight collections from 7 May through 28 September 2001, nine collections from 4 April through 20 September 2002) and approximately once monthly during the nonspawning season. The US2 and DS2 sites were sampled once monthly during the spawning season (four collections in 2001, six collections in 2002). Four to 14 individuals of each sex were sampled at each site on each date. Because of small sample size, collections from several dates during the 2001 spawning season were combined on the basis of Julian date and similarity of water quality. Data are plotted versus the median date of the combined sample periods. All other collections were made on the exact dates shown.

Captured fish were anesthetized (1:100,000 MS-222; Sigma-Aldrich, St. Louis, MO), bled (within 5 ± 0.003 min), measured, and marked with an individual-specific pattern of colored elastomer (Northwest Marine Technology, Shaw Island, WA). Blood was drawn from the caudal vein (27 gauge needle, 1 mL syringe with 6 mg/mL ammonium heparin in 0.9% NaCl) and mixed with 0.84 TIU/mL aprotinin, and then held on ice until centrifugation at 14,000 rpm for 5 min at 4°C. Plasma was aspirated and stored at –80°C. During the breeding season, gametes were expressed to determine sex. From late August 2001 through April 2002, fish were sacrificed for macroscopic examination of the gonads.

### Steroid analysis.

We analyzed 17β-estradiol (E_2_), T, and 11-ketotestosterone (11KT) by acetylcholinesterase-based competitive enzyme-linked immunosorbent assay (ELISA) performed according to manufacturer’s instructions (Cayman Chemical, Ann Arbor, MI). Before analysis, duplicate plasma aliquots (T and 11KT, 2 μL; E_2_, 15–25 μL) were triple extracted with anhydrous diethyl ether, evaporated to dryness, and reconstituted in assay buffer (1 M sodium phosphate, pH 7.4, 1% bovine serum albumin, 4 M NaCl, 10 mM EDTA, 0.1% sodium azide).

Parallel dilution of endogenous steroid in longear sunfish plasma relative to steroid standards was demonstrated for all steroids. Recovery of known steroid concentrations was 92% for T, 76.2% for E_2_, and 73% for 11KT. Intraassay variation was 13.2% ± 1.6 for T, 13.4% ± 2.2 for E_2_, and 8.1% ± 0.8 for 11KT. Interassay variation was 21.8% for T (*n* = 37 assays), 26.7% for E_2_ (*n* = 35 assays), and 19.9% for 11KT (*n* = 32 assays).

### VTG ELISA.

We analyzed VTG using a heterologous competitive ELISA developed for bluegill, *Lepomis macrochirus* (Cheek et al. 2004). Western blotting verified that anti-bluegill VTG antiserum recognized a single major polypeptide in plasma from E_2_-injected juvenile and vitellogenic female longear sunfish. Antisera were diluted 1:120,000, and longear sunfish plasma was diluted 1:9,000–1:6,000. Plasma from vitellogenic female longear sunfish diluted in parallel with the purified bluegill VTG standard curve. The average recovery of purified bluegill VTG in longear sunfish plasma was 84%; intraassay variation was 10.7%, and interassay variation was 17.4% (*n* = 37 assays).

### Statistics.

We analyzed site-specific differences in water quality using the Friedman test. Hormone values were log(10*Y* + 1) transformed (Sokal and Rohlf 1981). Two levels of analysis were performed. First, a two-way analysis of covariance for the main effects of date and site with size as a covariate was performed on the US1 and DS1 combined data set (2001–2002) to demonstrate seasonal patterns in reproductive physiology and evaluate site-specific changes. Size was included as a covariate because male androgen levels can increase with body size in species with alternative male reproductive strategies (Brantley et al. 1993; Kindler et al. 1989). If the interactions between size and main effects were not significant, the interaction terms were removed. If hormone or VTG levels did not vary significantly with size, the covariate was removed and a two-way analysis of variance for the main effects of site and date was performed. Second, because effluent concentration was twice as high during summer 2002 (Table 1), the data set was divided into 2001 and 2002 spawning seasons and the same analyses were performed.

## Results

### Water quality.

Although temperature profiles were similar among sites, temperatures differed significantly between sites (χ^2^ = 11.6, *p* = 0.02) with DS2 > DS1 > US1 > outfall > US2. This rank ordering is an artifact of sampling order: sites were sampled from upstream to downstream beginning at the farthest upstream location, US2, in the early morning when surface water temperature was coolest. Dissolved oxygen levels did not vary significantly between sites (χ^2^ = 8.86, *p* = 0.06), and all sites were always ≥ 69% saturation. Conductivity was slightly but significantly higher at downstream sites (χ^2^ = 29.036, *p* < 0.0001), showing an effluent signature.

### Effluent dilution.

Mean daily discharge of paper mill effluent was similar between spawning seasons (2001, 0.81 m^3^/sec; 2002, 0.94 m^3^/sec), but river flow was greater during the 2001 spawning season (234.2 m^3^/sec) than during the 2002 season (149.3 m^3^/sec; Table 1). Consequently, effluent concentration was higher during the 2002 spawning season than during the 2001 spawning season (0.95% vs. 0.45%; Table 1).

### Site fidelity.

A total of 697 longear sunfish were marked between October 2000 and August 2002. Of these, 18 (2.6%) were recaptured. Most recaptures occurred at the site of initial capture (83%). No marked fish migrated from upstream (reference) to downstream (effluent receiving), but one moved from downstream to upstream, one migrated from one downstream site to the other, and one moved from one upstream site to the other.

### Reproductive physiology.

#### Males.

**Figure 2:**
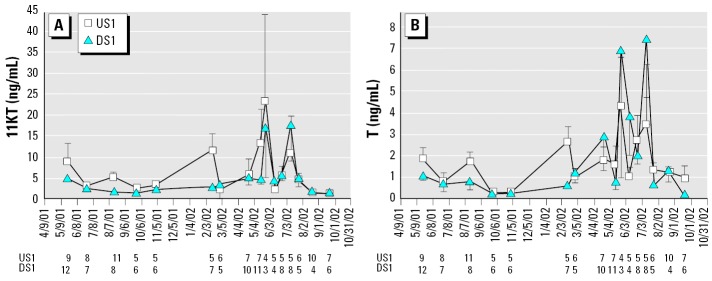
Male longear sunfish sampled from the Pearl River near Bogalusa, Louisiana. (*A*) 11KT. (*B*) T. Numbers below the *x*-axis indicate sample sizes for each site on each date. Both androgens increased with body size and varied significantly over time, but did not differ between sites. Values are mean ± SEM.

Male androgen levels increased significantly with body size (T: *F*_size_ = 46.4, *p* < 0.0001; 11KT: *F*_size_ = 56.01, *p* < 0.0001) and varied significantly over time (11KT: *F*_date_ = 4.10, *p* < 0.0001; T: *F*_date_ = 2.4, *p* = 0.0049) but were similar between sites (Figure 2). Separate analysis of the two spawning seasons gave the same results. Intensive sampling during 2002 showed that T and 11KT peaked twice during the spawning season (T: *F*_date_ = 2.77, *p* = 0.01; 11KT: *F*_date_ = 4.28, *p* = 0.0002).

**Figure 3:**
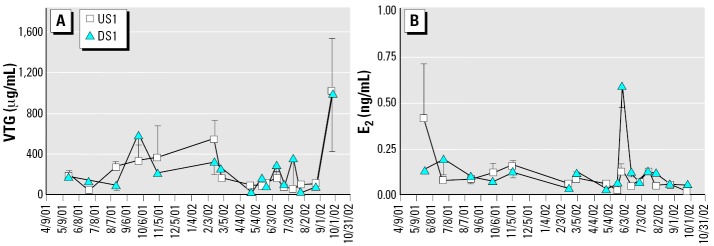
Male longear sunfish sampled from the Pearl River near Bogalusa, Louisiana. (*A*) VTG. (*B*) E_2_. Neither VTG nor E_2_ varied with body size or between sites, but both parameters showed significant seasonal variation. Sample sizes for each site on each date are shown in Figure 2B. Values are mean ± SEM.

E_2_ and VTG levels were similar among all sizes of males but varied significantly over time (Figure 3; E_2_: *F*_date_ = 3.31, *p* < 0.0001; VTG: *F*_date_ = 3.93, *p* < 0.0001). Separate analysis of the two spawning seasons showed that a single significant increase in E_2_ occurred during late May 2002 at DS1 (*F*_site_ = 54.62, *p* < 0.0001; *F*_date_ = 27.95, *p* < 0.0001; *F*_site_ × date = 13.62, *p* < 0.0001). Peak E_2_ production coincided with peak T and 11KT production at both sites. In contrast, male VTG concentrations were significantly higher during the nonspawning season, October through March, with a marked increase occurring in September at both sites (Figure 3; 2001: *F*_date_ = 6.05, *p* = 0.001; 2002: *F*_date_ = 4.16, *p* = 0.0003).

#### Females.

**Figure 4:**
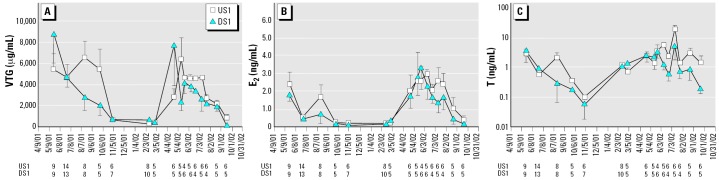
Female longear sunfish sampled from the Pearl River near Bogalusa, Louisiana. (*A*) VTG. (*B*) E_2_. (*C*) T (note logarithmic scale). All three parameters varied significantly over time. Numbers below the *x*-axis indicate sample sizes for each site on each date. E_2_ was similar between sites; VTG was significantly lower at the downstream site in 2002 when effluent concentration exceeded 1% of river flow; and T was significantly lower in females sampled at the downstream site. Values are mean ± SEM.

All sizes of females had similar VTG levels, and VTG changed significantly over time (combined 2001 and 2002 data, *F*_date_ = 5.80, *p* < 0.0001), with the highest concentrations during the spawning season (Figure 4A). The effects of site and date differed between years. In 2001, average VTG concentration was statistically similar between sites. In contrast, during 2002, VTG increased sharply between early March and late April at the upstream site and remained elevated through late July. At the effluent-receiving site, VTG also increased sharply in March and April but then declined steadily throughout the summer (Figure 4A; *F*_date_ = 6.3, *p* < 0.0001; *F*_site_ × date = 3.28, *p* = 0.003).

Female E_2_ and T increased significantly with body size and varied significantly over time. The highest hormone concentrations occurred during the spawning season (Figure 4B, C; E_2_: *F*_date_ = 11.82, *p* < 0.0001; T: *F*_date_ = 7.10, *p* < 0.0001) and were similar between sites (2001: *F*_date_ = 8.83, *p* < 0.0001; 2002: *F*_date_ = 4.78, *p* = 0.0001). Female T concentrations varied significantly throughout the spawning season in both years (2001: *F*_date_ = 3.21, *p* = 0.03; 2002: *F*_date_ = 4.58, *p* = 0.0002). Average T concentration was significantly lower at the effluent-receiving site in the combined data set (2001 and 2002) and in 2002 (Figure 4C; combined 2001 and 2002: *F*_site_ = 5.2, *p* = 0.02; 2002: *F*_site_ = 7.97, *p* = 0.0062).

**Figure 5:**
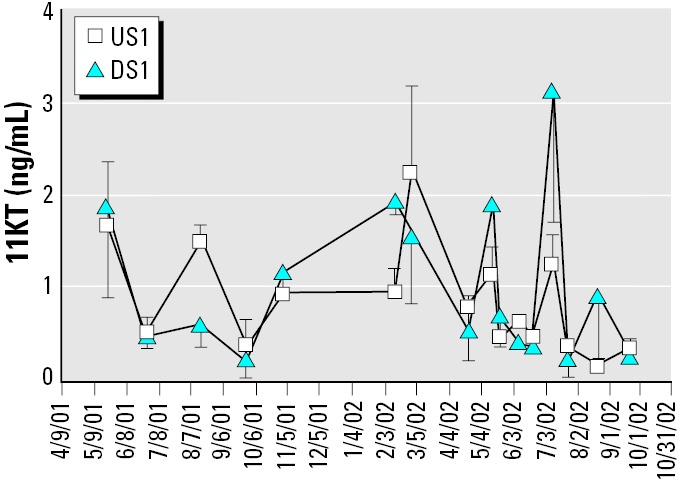
Female longear sunfish sampled from the Pearl River near Bogalusa, Louisiana. 11KT varied significantly over time, but not between sites. Sample sizes for each site on each date are shown in Figure 4C. Values are mean ± SEM.

Female 11KT was unrelated to body size and did not differ between sites but did change throughout the year (Figure 5; *F*_date_ = 6.14, *p* < 0.0001). Unlike E_2_, T, and VTG, female 11KT values were elevated during the non-spawning season, October through March.

At the sandbar sites, US2 and DS2, the relationships of male and female hormone and VTG concentrations to sampling date and body size were similar to those observed at the streamside sites (US1 and DS1; data not shown). Although data from the sandbar sites generally corroborate data from the streamside sites, far fewer individuals were sampled at the sandbar sites (*n* = 42 females and 57 males upstream, *n* = 38 females and 51 males at the effluent receiving site) compared with the streamside sites (*n* = 104 females and 105 males upstream and 102 females and 110 males at the effluent receiving site). Also, the second effluent-receiving site (DS2) was 0.3 km farther downstream, creating the possibility of greater effluent dilution compared with DS1.

## Discussion

Establishing potential causal relationships between contaminant sources and effects in free-living animals is always difficult because animals are exposed to multiple environmental stressors. One approach is to measure the contaminants of interest in the environment and in animal tissues and then suggest causal relationships based on the concentrations measured. The limitation of this approach is that only a single possible cause of the effect has been identified and quantified (Adams et al. 1996).

We used an approach that evaluates the likelihood of exposure to an industrial effluent based on fish movement patterns. We marked 697 longear sunfish over a 2-year period and recaptured 2.6% of marked fish. Our recapture rate in a large, fast-flowing river is low compared with recapture rates in small streams (Berra and Gunning 1972; Smithson and Johnston 1999); however, the percentage of recaptured fish remaining at the site of initial capture is almost identical. In our study, 83% of recaptured fish were found at the site of initial capture. Previous investigations also indicated limited adult migration, 70–90% of recaptured longear sunfish were found at the site of initial capture (Berra and Gunning 1972; Gunning and Shoop 1963; Smithson and Johnston 1999). Because adults exhibit strong site fidelity in the Pearl River, we can reasonably assume that longear sunfish captured at effluent receiving sites are residents, not transitory migrants.

Unbleached KME from the Bogalusa Mill appears to have little effect on male longear sunfish reproductive physiology. Androgen levels did not vary between sites, nor did E_2_ or VTG levels. Regardless of effluent exposure, androgen concentrations increased with male body size and varied significantly throughout the year and the spawning season. More frequent sampling during the 2002 spawning season clearly documented multiple T and 11KT peaks (Figure 2). High plasma androgen levels are probably associated with the initiation of spawning bouts as they are in bluegill, a closely related species with similar reproductive strategies (Kindler et al. 1989).

Although KME exposure had no effect, male E_2_ and VTG showed significant seasonal variation (Figure 3). Mean plasma E_2_ was 0.11 ng/mL in males, a value approximately 10-fold lower than the overall mean in females (1.56 ng/mL). Male VTG concentrations increased sharply in late September and were highest during the nonspawning period (Figure 3). The average fall/winter VTG concentration in males (342 μg/mL) was equivalent to the average concentration in females during the same period (454 μg/mL) but was 10-fold lower than the spawning female average (4,400 μg/mL).

Much recent work has promulgated the paradigm that normal male fish produce little E_2_ and undetectable amounts of VTG. When investigators detect VTG in reference or control males, the findings are often labeled “unexpected” and are sometimes attributed to a prior, unknown exposure to an estrogenic substance (Denslow et al. 2004). We suggest that low circulating levels of E_2_ and VTG are part of normal male fish physiology.

Male E_2_ levels have been measured infrequently in wild fish, but seasonal differences have been documented in perch, roach (Karels et al. 1998, 2001), cutthroat trout (Fukada et al. 2001), plainfin midshipman (Sisneros et al. 2004), and longear sunfish (the present study). E_2_ concentrations were highest before the single annual spawning event in male roach, cutthroat trout, and perch (Fukada et al. 2001; Karels et al. 1998, 2001). In males that spawn multiple times during an extended reproductive season, E_2_ levels may be low and constant throughout the year as in male midshipman (Sisneros et al. 2004) or may cycle during the spawning season as in male longear sunfish (the present study). Given the important role of E_2_ in mammalian spermatogenesis (Couse and Korach 1999), the presence of estrogen receptor (ER)-α in the fish testis (Bouma and Nagler 2001), and the seasonality of circulating E_2_ in a variety of wild male fish, low but detectable E_2_ in males from reference or control populations should be considered normal.

Likewise, low concentrations of VTG in unexposed male fish are probably normal. Seasonal variation in VTG occurs in wild male cutthroat trout (Fukada et al. 2001), roach (Karels et al. 2001), and longear sunfish (the present study) collected from relatively pristine locations. Male VTG concentrations vary between species, ranging from 5–200 ng/mL in cutthroat trout, roach, and mummichog to several hundred micrograms per milliliter in largemouth bass, longear sunfish, and cunner (*Tautogolabrus adspersus*) (the present study; Hiramatsu et al. 2005).

The function of VTG in male fish is an intriguing question. Perhaps it is produced as a physiologic artifact in response to low levels of endogenous E_2_ (Hiramatsu et al. 2005). We suggest that VTG could serve an osmo-regulatory role. Ion loss across the body surface, particularly the gills, is a continual osmotic challenge for freshwater and estuarine fish. Calcium is one of the major ions actively transported across the gill epithelium into the bloodstream of freshwater fishes (Perry 1997). VTG strongly binds calcium (Ng and Idler 1983) and could serve as a plasma calcium reservoir. Consistent with this hypothesis, VTG increased in male longear sunfish in the fall, a time when river flow increased and conductivity (ion concentration) decreased. Decreased ion concentration creates a more pronounced osmotic gradient between the fish and its environment. An osmotic function would also be consistent with Kirby et al.’s (2004) observation that VTG concentrations increase in male flounder (*Platichthys flesus*) from fall through spring—a period when they migrate from the coastal shelf into estuaries.

Unbleached KME suppressed vitellogenesis in female longear sunfish, but the effect occurred only in 2002 when effluent constituted ≥ 1% of river flow for 3 months (May through July) and averaged 0.95% of flow from May through September (Table 1). No alteration of female T, E_2_, or VTG occurred during 2001 when effluent constituted 0.45% of flow from May through September and never exceeded 1% (Table 1).

What are the consequences of suppressed VTG production? Females may release fewer eggs per spawning bout or may spawn fewer times during the reproductive season. Based on the number of sampling periods with elevated T concentrations, females at the effluent receiving site appear to have spawned fewer times in both years—twice upstream versus once downstream in 2001 and three times upstream versus twice downstream in 2002 (Figure 4).

Oddly, VTG suppression in KME-exposed female longear sunfish occurred without concurrent E_2_ suppression. Given that E_2_ specifically stimulates vitellogenesis in the liver and that T is the precursor for ovarian E_2_ production, one would predict that suppressed VTG would be accompanied by suppressed E_2_ and possibly T. Instead, E_2_ was unaffected, although T and VTG were significantly reduced. Apparently, sufficient T exists to allow unaltered E_2_ production. Why is VTG suppressed? Perhaps T up-regulates the number of ER in the liver, indirectly enhancing sensitivity to E_2_ stimulation. With suppressed T concentrations, perhaps ER sensitivity is reduced, resulting in slightly depressed VTG production. A few reports of *in vivo* T treatment enhancing estrogen-binding capacity in rodents lend some support to this idea (Cano et al. 1986; Ho and Yu 1993; Jaubert et al. 1995).

Although female T was suppressed at the KME-receiving site, female 11KT was not different between sites. The function of 11KT in female fish is unknown (Lokman et al. 2002), but in longear sunfish, the female and male 11KT profiles coincided, suggesting that 11KT may play a role in female spawning behavior. Average female 11KT (1.47 ng/mL) was 5-fold lower than average male 11KT. In contrast, average female T concentration during the spawning season (3.98 ng/mL) exceeded male T (1.99 ng/mL). T concentrations in vitellogenic females often equal or exceed T concentrations in spermiating males of many fish species (Lokman et al. 2002), possibly because of the critical role of T as a precursor for E_2_.

Reproductive suppression occurs in fish downstream from paper mills regardless of bleaching or secondary treatment processes used (Dube and MacLatchy 2000; Karels et al. 2001; McMaster et al. 1996; Sepulveda et al. 2002). Significant hormonal effects occur when mill effluents constitute ≥ 1% (volume) of the receiving environment (Dube and MacLatchy 2000; Sepulveda et al. 2002). The Bogalusa Mill is an elemental chlorine-free mill with secondary treatment, but longear sunfish in the receiving stream experience reproductive suppression, particularly when effluent flow equals or exceeds 1% of river flow. Mill processes are unlikely to explain this suppression, nor is climate or habitat: reproductive suppression occurs in both cold (Canada and Finland) and warm (Florida and Louisiana, USA) climates and in fresh and saltwater (Dube and MacLatchy 2000; Karels et al. 2001; Sepulveda et al. 2002). Organic compounds in the wood itself are the most likely cause of reproductive suppression (Dube and MacLatchy 2001). Hewitt et al. (2002) showed that compounds derived from both hardwoods and softwoods caused similar suppressive effects.

## Conclusions

Longear sunfish in the Pearl River experience moderate reproductive suppression in response to unbleached KME. Males appear to be unaffected, but females experience suppressed vitellogenesis when KME constitutes ≥ 1% of river flow. No alterations in male or female androgen status or secondary sex characteristics were observed, indicating that unbleached KME from the Bogalusa Mill does not masculinize sunfish. Modulating effluent flow in response to seasonal and annual changes in river flow could maintain effluent concentrations below 1% in receiving water and minimize reproductive impacts on sunfish.

## Figures and Tables

**Table 1:** Mean daily flow rate of the Pearl River at Bogalusa, Louisiana, and mean daily wastewater discharge from the Bogalusa Mill aeration basin.

Month	Pearl River^a^ (m^3^/sec)	Bogalusa mill^b^ (m^3^/sec)	Effluent (% river flow)
Oct 2000	38	0.93	2.44
Nov 2000	87	0.93	1.07
Dec 2000	83	1.06	1.28
Jan 2001	360	0.84	0.23
Feb 2001	277	0.92	0.33
Mar 2001	1,026	0.90	0.09
Apr 2001	344	0.82	0.24
May 2001	88	0.83	0.95
Jun 2001	192	0.87	0.45
Jul 2001	138	0.81	0.58
Aug 2001	296	0.82	0.28
Sep 2001	346	0.73	0.21
Oct 2001	190	0.75	0.39
Nov 2001	82	0.82	1.00
Dec 2001	451	0.80	0.18
Jan 2002	315	NR	
Feb 2002	523	0.79	0.15
Mar 2002	364	0.90	0.25
Apr 2002	428	0.95	0.22
May 2002	71	0.89	1.25
Jun 2002	65	0.98	1.51
Jul 2002	74	0.92	1.23
Aug 2002	128	0.89	0.69
Sep 2002	131	1.01	0.77
Oct 2002	401	0.92	0.23

NR, no report filed with the LDEQ.

a Measured at site USGS 02489500, 30°47′35″ N, 89°49′15″ W, Pearl River near Bogalusa, Louisiana. Data from USGS (2003).

b As reported in monthly discharge monitoring reports to the LDEQ (2002).
